# The yeast cell wall protein Pry3 inhibits mating through highly conserved residues within the CAP domain

**DOI:** 10.1242/bio.053470

**Published:** 2020-07-03

**Authors:** Stéphanie Cottier, Rabih Darwiche, Felix Meyenhofer, Mykhaylo O. Debelyy, Roger Schneiter

**Affiliations:** Department of Biology, University of Fribourg, Chemin du Musée 10, 1700 Fribourg, Switzerland

**Keywords:** CAP protein family, Fatty acids, Sterols, GPI-anchored proteins, Mating, Cell wall, *Saccharomyces cerevisiae*

## Abstract

Members of the CAP/SCP/TAPS superfamily have been implicated in many different physiological processes, including pathogen defense, sperm maturation and fertilization. The mode of action of this class of proteins, however, remains poorly understood. The genome of *Saccharomyces cerevisiae* encodes three CAP superfamily members, Pry1-3. We have previously shown that Pry1 function is required for the secretion of sterols and fatty acids. Here, we analyze the function of Pry3, a GPI-anchored cell wall protein. Overexpression of Pry3 results in strong reduction of mating efficiency, providing for a cell-based readout for CAP protein function. Mating inhibition is a conserved function of the CAP domain and depends on highly conserved surface exposed residues that form part of a putative catalytic metal-ion binding site. Pry3 displays polarized cell surface localization adjacent to bud scars, but is absent from mating projections. When overexpressed, however, the protein leaks onto mating projections, suggesting that mating inhibition is due to mislocalization of the protein. Trapping of the CAP domain within the cell wall through a GPI-anchored nanobody results in a dose-dependent inhibition of mating, suggesting that a membrane proximal CAP domain inhibits a key step in the mating reaction, which is possibly related to the function of CAP domain proteins in mammalian fertilization.

This article has an associated First Person interview with the first author of the paper.

## Abstract (precis)

**Summary:** Overexpression of Pry3, a GPI-anchored cell wall protein inhibits the yeast mating reaction. Here we show that this mating inhibition depends on highly conserved residues within the CAP/SCP/TAPS domain of Pry3.

## INTRODUCTION

Proteins belonging to the CAP superfamily (cysteine-rich secretory proteins, antigen 5, and pathogenesis related 1 proteins; Pfam PF00188), also known as sperm coating proteins (SCP), TAPs (Tpx, antigen 5, pathogenesis-related 1), or venom allergen-like proteins (VALs), are present in all kingdoms of life and have been implicated in many different physiological processes, including immune defense in mammals and plants, pathogen virulence, sperm maturation and fertilization, venom toxicity, as well as prostate and brain cancer. Most CAP proteins are secreted glycoproteins and they all share a common CAP domain of approximately 150 amino acids, which adopts a unique α−β−α sandwich fold, wherein the central β-sheet is flanked by three helices on one side and a fourth helix on the other side. The structural conservation of this domain suggests that CAP proteins exert a fundamentally similar molecular function. The precise mode of action of this class of proteins, however, has remained elusive (for reviews see Breen et al., 2017, Cantacessi et al., 2009, Gibbs et al., 2008, Osman et al., 2012, Schneiter and Di Pietro, 2013, Wilbers et al., 2018).

Several CAP family members have been shown to bind lipids, suggesting that lipid-binding may constitute a conserved mode of action of these proteins. For example, the smallest of the mammalian CAPs, glioma pathogenesis-related 2 protein (GLIPR-2/RTVP-1/GAPR-1) interacts with the surface of liposomes containing negatively charged lipids and binds phosphatidylinositol (Eberle et al., 2002; Van Galen et al., 2010). Moreover, GAPR-1 binds Beclin-1, a key autophagy protein, and downregulates autophagy (Shoji-Kawata et al., 2013). On the other hand, tablysin-15, a salivary venom allergen of the blood-feeding horsefly, *Tabanus yao*, which acts as a potent inhibitor of platelet aggregation, binds eicosanoids as well as free fatty acids, thereby inhibiting the proinflammatory action of cysteinyl leukotrienes released from mast cells in the area of the insect bite (Xu et al., 2012).

CAP superfamily members from *Saccharomyces cerevisiae*, termed pathogen related in yeast (Pry proteins), promote the export of sterols and fatty acids *in vivo* and purified Pry1 binds these two lipids in distinct non-overlapping binding sites *in vitro* (Choudhary and Schneiter, 2012; Darwiche et al., 2017b). The fatty acid- and sterol-binding sites are both confined to the conserved CAP domain and CAP family members from other species have also been shown to bind sterols and fatty acids (Darwiche et al., 2016, 2017a,b; Gamir et al., 2017; Kelleher et al., 2014). These results indicate that lipid-binding and sequestration may constitute a shared mode of action of CAP family members (Breen et al., 2017; Darwiche et al., 2018; Kazan and Gardiner, 2017).

Apart from lipid binding, CAP proteins have also been proposed to harbor proteolytic activity. Tex31, a CAP family member from a cone snail, was purified based on its protease activity (Milne et al., 2003). Homology modeling revealed that the likely catalytic residues of Tex31 were located in a structurally conserved domain of CAP proteins, resembling that of serine proteases, suggesting that CAP proteins may act as substrate-specific proteases (Milne et al., 2003).

Here, we analyze the mode of action of Pry3, a CAP family member from *S. cerevisiae* that is associated with the cell wall and contains a predicted C-terminal glycosylphosphatidylinositol (GPI)-anchor (Eisenhaber et al., 2004; Hamada et al., 1998; Yin et al., 2005). Interestingly, synthesis of full-length Pry3 is repressed in the presence of mating pheromone and overexpression of Pry3 results in a strongly decreased mating efficiency (Bickel and Morris, 2006). In the present study, we show that mating inhibition is a functionally conserved feature of the CAP domain that is independent of its lipid-binding activity, but requires highly conserved, surface-exposed residues. Pry3 displays polarized cell surface localization adjacent to bud scars. Upon overexpression, the protein is more uniformly distributed, suggesting that mating inhibition could be due to mislocalization of the protein, particularly its presence on polarized mating projections. Consistent with this proposition, fusion of the CAP domain to a cell wall protein that is localized to mating projections, Ccw12, results in mating inhibition. These results suggest that the function of the yeast CAP domain in mating inhibition may be related to the function of CAP domain-containing CRISP proteins in sperm maturation and fertilization.

## RESULTS AND DISCUSSION

### Mating inhibition by overexpression of Pry3 requires the GPI-anchored CAP domain but not the serine/threonine-rich region

**Fig. 1.**
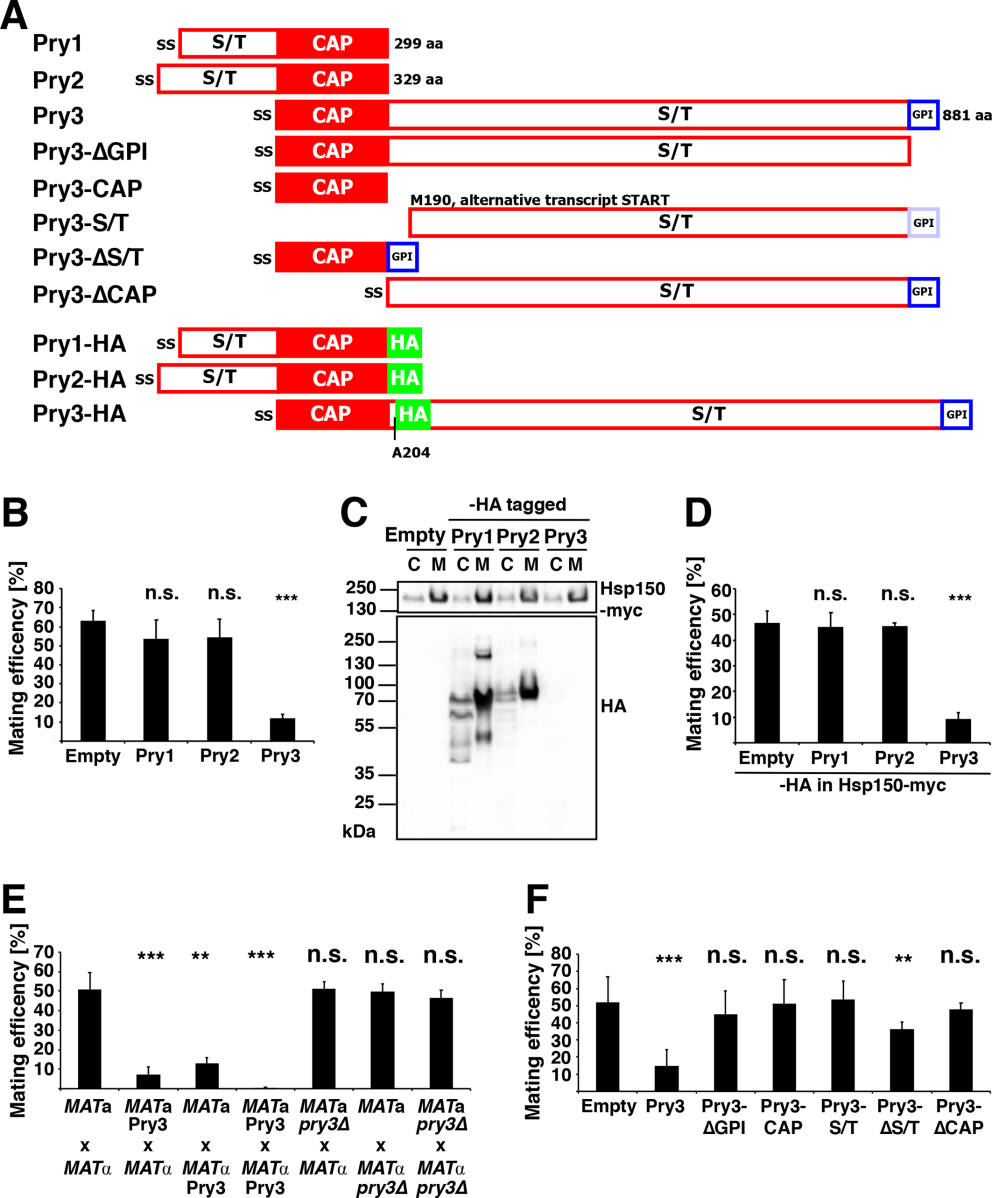
**The CAP domain of Pry3 is required for mating inhibition.** (A) Domain structure of the yeast CAP family members and schematic illustration of the truncated versions of Pry3 tested for their ability to confer mating inhibition. The three yeast CAP family members Pry1, Pry2 and Pry3 are composed of a conserved CAP domain (red box) and a serine/threonine-rich domain (S/T) of varying length. All three proteins enter the secretory pathway due to the presence of an N-terminal signal sequence (ss). Pry3 is unusual in that it contains the serine/threonine-rich region downstream of the CAP domain and the protein is GPI-anchored (blue box). The GPI-anchor of Pry3-S/T is highlighted in light blue, since the sequence should not be recognized as GPI-anchor due to the absence of the signal sequence. Deletion constructs of Pry3 tested for functionality in a mating assay, Pry3-ΔGPI, Pry3-CAP, Pry3-S/T, Pry3-ΔS/T and Pry3-ΔCAP are depicted. The positions of the HA tags at the C-termini of Pry1 and 2 and the internal tag in Pry3, placed after alanine 204, are represented by green boxes. (B) Among the yeast CAP proteins, only Pry3 overexpression inhibits mating. Cells transformed with an empty plasmid or overexpressing either Pry1, Pry2 or Pry3 were mated to wild-type cells of the opposite mating type for 5 h at 30°C. Cells were then plated on solid media and mating efficiency was quantified and plotted. (C) Pry1 and Pry2 are secreted glycoproteins, Pry3 is not detectable by western blotting. Protein extracts from total cells (C) and culture media (M) expressing HA-tagged Pry1, Pry2 and Pry3 were analyzed by western blotting. Detection of the secreted Hsp150-myc serves as secretion control. (D) HA-tagged Pry3 is functional in mating inhibition. Mating efficiency of cells transformed with an empty plasmid or overexpressing either HA-tagged Pry1, Pry2 or Pry3. (E) Mating inhibition by Pry3 is additive. Cells of the indicated mating types either overexpressing (Pry3) or lacking Pry3 (*pry3*Δ) were tested for mating efficiency. (F) The GPI-anchored CAP domain of Pry3 is sufficient for mating inhibition. Mating efficiency of cells containing an empty plasmid, overexpressing wild-type Pry3, or the indicated deletion variants of Pry3. Values for mating efficiency shown in panels B, D, E and F represent means±s.d. of four independent determinations. Asterisks denote statistical significance (Welch *t*-test; **P-value <0.01; ***P-value <0.001; n.s., not significant).

Yeast expresses three members of the CAP superfamily, Pry1, Pry2 and Pry3. These secreted glycoproteins contain a CAP domain of roughly 150 amino acids and either an N-terminal (Pry1, Pry2) or C-terminal serine/threonine-rich region (Fig. 1A) (Choudhary and Schneiter, 2012; Darwiche et al., 2018). The serine/threonine-rich region is particularly long in Pry3, covering more than 600 amino acids. In addition, Pry3 is unusual in that it contains a predicted consensus sequences for attachment of a GPI-anchor (Eisenhaber et al., 2004; Hamada et al., 1999). Proteomic studies found Pry3 to be associated with the yeast cell wall rather than being secreted into the culture media as is the case for Pry1 and Pry2 (Choudhary and Schneiter, 2012; Hamada et al., 1998; Yin et al., 2005). *pry3*Δ mutant cells do not exhibit any particular phenotype; however, overexpression of *PRY3* results in a strong inhibition of the mating reaction (Bickel and Morris, 2006). While the precise molecular mechanism that accounts for this mating inhibition by Pry3 is presently unknown, we used mating efficiency in this study as a cell-based readout to further characterize the function of CAP domain containing proteins. Mutant cells lacking *pry3*Δ or cells overexpressing *PRY3* show no particular growth phenotype and overexpression of Pry3 does not affect agglutination of cells during mating.

To test whether mating inhibition is specific for Pry3 or whether this could also be observed upon overexpression of Pry1 or Pry2, *MAT***a** cells were transformed with plasmids overexpressing these Pry proteins, cells were mated to *MAT*α cells for 5 h at 30°C and the number of zygotes formed during this period of time was quantified. While overexpression of Pry3 resulted in a significant inhibition of the mating reaction, no mating inhibition was observed upon overexpression of the secreted Pry1 or Pry2 members of this CAP family (Fig. 1B). Western blotting confirmed that hemagglutinin (HA)-tagged versions of Pry1 and Pry2 were secreted as high molecular weight glycoproteins into the culture media as was a myc-tagged version of a secreted heat shock protein, Hsp150 (Fig. 1C). Under these conditions, however, an internally HA-tagged version of Pry3 could not be detected by western blotting, probably because the protein is both highly glycosylated and covalently attached to the cell wall. However, the HA-tagged versions of Pry3 was functional in mating inhibition indicating that the protein is properly expressed (Fig. 1D).

Mating inhibition by Pry3 was independent of the mating type in which the protein was expressed but the inhibition was enhanced when the protein was overexpressed in both mating partners (Fig. 1E). Deletion of Pry3, on the other hand, did not affect mating efficiency (Fig. 1E).

To understand which domains of Pry3 are required for mating inhibition, we generated truncated versions of Pry3 lacking either the GPI-attachment site (Pry3-ΔGPI), the CAP domain (Pry3-ΔCAP), or the serine/threonine-rich region (Pry3-ΔS/T), as well as versions expressing the CAP domain alone (Pry3-CAP), or only the serine/threonine-rich region (Pry3-S/T) (Fig. 1A). The truncated version of Pry3 containing only the serine/threonine-rich region, Pry3-S/T, but lacking the CAP domain, is predicted to represent a natural variant of Pry3, derived from an alternative, shortened transcript that is specifically generated upon exposure of cells to mating pheromone (Bickel and Morris, 2006). Analysis of these truncated variants of Pry3 in the quantitative mating assay revealed that mating inhibition required the presence of both the GPI-attachment site and the CAP domain (Fig. 1F). The serine/threonine-rich region of Pry3, on the other hand, was not essential, because its expression either in form of Pry3-ΔCAP, or in form of the shortened, mating-specific transcript that generates Pry3-S/T, did not affect mating (Fig. 1F). Taken together, these data indicate that the soluble secreted CAP domain of Pry3 is not sufficient to inhibit mating, but that the GPI-anchored CAP domain is both necessary and sufficient to inhibit the mating reaction. Presence of the S/T region on the other hand slightly enhances the impact of the CAP domain on mating inhibition.

### Mating inhibition is a conserved function of the yeast CAP domain

**Fig. 2.**
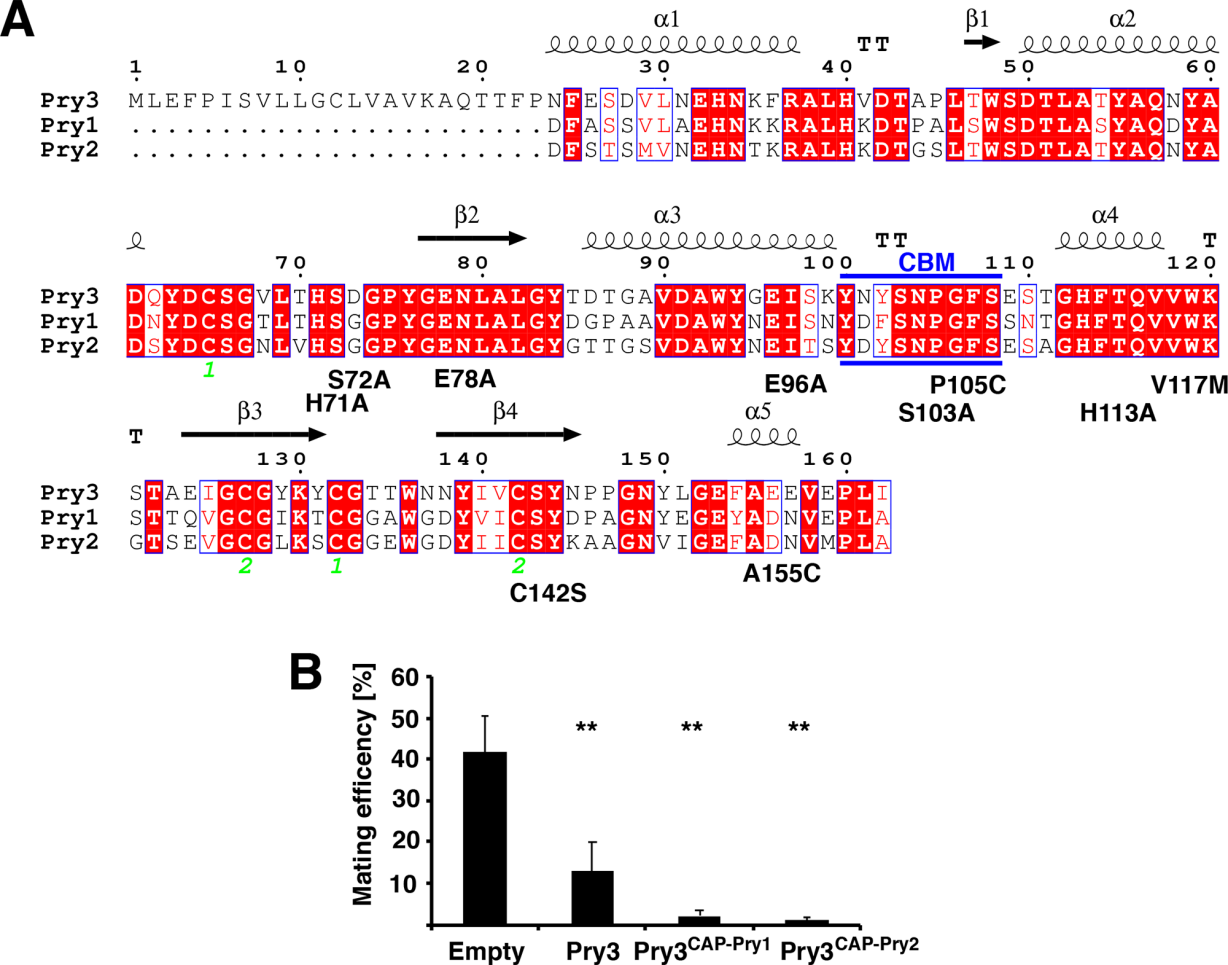
**Mating inhibition is a conserved function of the yeast CAP domain.** (A) The CAP domain of Pry3 is highly homologous to that of Pry1 and Pry2. Sequence alignment of the CAP domain from yeast Pry3 to that of Pry1 and Pry2 was generated with ESPrit 3.0 (Robert and Gouet, 2014). Secondary structure elements are indicated above the sequences, conserved residues are boxed red. Green numbers indicate disulfide bridges. Blue lines highlight the position of the Caveolin-Binding Motif (CBM). Mutations introduced into Pry3 CAP domain in this study are indicated below the alignment. (B) Variants of Pry3 containing the CAP domain of either Pry1 or Pry2 are functional in inhibiting mating. Mating efficiency of cells containing an empty plasmid, overexpressing a wild-type version of Pry3, or the indicated variants of Pry3 containing the CAP domain of either Pry1, Pry3^CAP-Pry1^ or Pry2, Pry3^CAP-Pry2^. Values represent means±s.d. of four independent determinations. Asterisks denote statistical significance (Welch *t*-test; **P-value <0.01;***P-value <0.001; n.s., not significant).

The CAP domain constitutes the defining feature of all CAP superfamily members. The domain adopts a unique α−β−α fold and has previously been shown to bind lipids, particularly eicosanoids, fatty acids, sterols and negatively charged lipids (Choudhary and Schneiter, 2012; Darwiche et al., 2017b; Van Galen et al., 2010; Xu et al., 2012). Sequence alignment of the CAP domain of Pry3 to that of Pry1 and Pry2 revealed that many of the key residues of Pry1, which have previously been shown to be required for sterol binding, such as cysteine 142, or binding of fatty acids, such as valine 117, are conserved in the CAP domain of Pry3 (Choudhary and Schneiter, 2012; Darwiche et al., 2017b) (Fig. 2A).

Given that functionally characterized key residues in the CAP domain are conserved between Pry1 and Pry3, and the fact that expression of the GPI-anchored CAP domain of Pry3 is sufficient for mating inhibition, we tested whether the CAP domain of Pry1 or that of Pry2 could substitute for the respective domain of Pry3 in mating inhibition. Therefore, we generated new versions of Pry3 in which the endogenous CAP domain was replaced either by the CAP domain of Pry1 or that of Pry2. Overexpression of both of these versions of Pry3, Pry3^CAP-Pry1^ or Pry3^CAP-Pry2^ resulted in strong mating inhibition, indicating that mating inhibition is not specific to the CAP domain of Pry3 but is a well-conserved function of the yeast CAP domain (Fig. 2B).

### The lipid-binding function of the CAP domain is not required for mating inhibition by Pry3

**Fig. 3.**
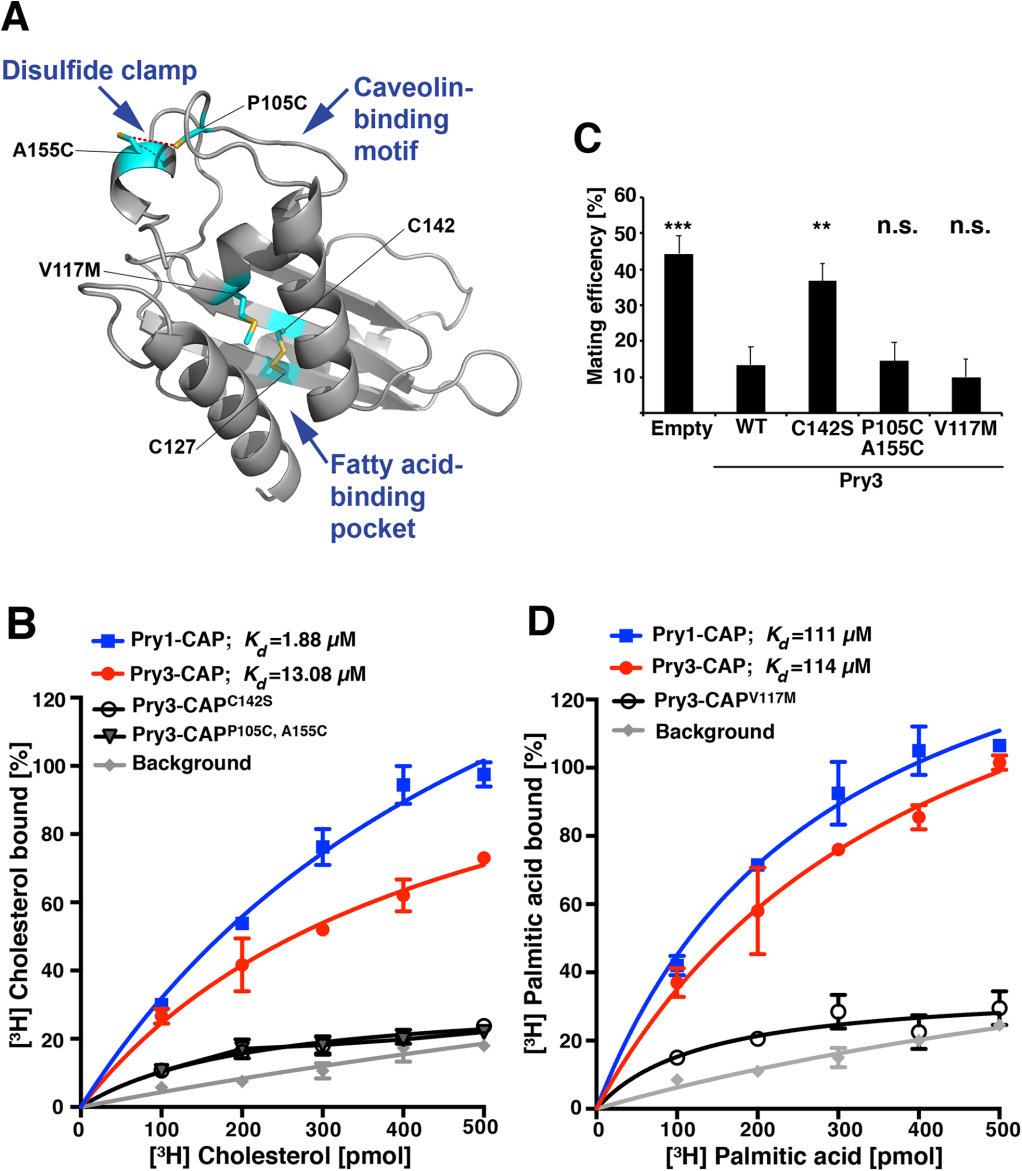
**Mating inhibition by Pry3 is independent of its lipid-binding function.** (A) Structural model of the CAP domain of Pry3 showing residues important for sterol- and fatty acid-binding. The simultaneous exchange of proline 105 and alanine 155 to cysteine (P105C, A155C) is expected to result in a disulfide bridge that clamps down the flexibility of the loop containing the caveolin-binding motif and thus to prevent sterol binding. Exchange of valine 117 to methionine obstructs the fatty acid binding pocket. Mutation of cysteine 142 to serine, will affect the disulfide bridge that connects two antiparallel β-sheets (β3 and β4). (B) The CAP domain of Pry3 binds cholesterol *in vitro* and this is abrogated by a mutation in the CAP domain, Pry3-CAP^P105C, A155C^. Polyhistidine-tagged versions of the CAP domain of Pry1, Pry3 and mutant versions of Pry3, Pry3-CAP^C142S^ and Pry3-CAP^P105C, A155C^ were expressed in *E. coli*, purified and binding of [^3^H]cholesterol was measured. Binding of the radioligand is plotted and the deduced *K_d_* is indicated. Background binding was determined in an assay lacking protein. (C) Mating inhibition by Pry3 is independent of the lipid-binding function of the CAP domain. Mating efficiency was calculated for cells containing an empty plasmid, a plasmid with a wild-type version of Pry3, or the mutant forms of Pry3: Pry3^C142S^, Pry3^P105C, A155C^, or Pry3^V117M^. Values represent means±s.d. of four independent determinations. Asterisks denote statistical significance compared to cells overexpressing wild-type (WT) Pry3 (Welch *t*-test; **P-value <0.01; ***P-value<0.001; n.s., not significant). (D) The CAP domain of Pry3 binds fatty acids *in vitro* and this binding is abrogated by the V117M mutation. Polyhistidine-tagged versions of the CAP domain of Pry1, Pry3 and the Pry3-CAP^V117M^ mutant version were purified from *E. coli*, and binding of [^3^H] palmitic acid was measured. The concentration-dependent binding of the radioligand is plotted and the *K_d_* is indicated. Values in panels B and D represent means±s.d. of two independent binding assays.

To identify residues within the CAP domain of Pry3 that are functionally important for mating inhibition, we generated a structural model of the CAP domain of Pry3 based on the crystal structure of the CAP domain of Pry1 (PDB ID: 5JYS) using HHpred (Darwiche et al., 2016; Hildebrand et al., 2009; Webb and Sali, 2017; Zimmermann et al., 2018). This analysis revealed spatial conservation of residues that have previously been shown to be important for sterol- and fatty acid-binding, respectively. In particular, the flexible loop rich in aromatic amino acids, harboring the so-called caveolin-binding motif, which is important for binding sterols, as well as the fatty acid-binding pocket between helices α1 and α3, are both conserved between Pry1 and Pry3 (Choudhary et al., 2014; Darwiche et al., 2017b; Xu et al., 2012) (Fig. 3A).

We have previously shown that CAP domain proteins export sterols from yeast cells *in vivo* and that the purified CAP domain binds sterols with micromolar affinity *in vitro* (Choudhary and Schneiter, 2012; Darwiche et al., 2016). Since sterols have important functions in membrane polarization and other aspects of the mating reaction, such as signal transduction, it is plausible that a sterol-binding activity of Pry3 could negatively impact the mating reaction (Aguilar et al., 2010; Jin et al., 2008). Sterol binding by the CAP domain requires a flexible loop rich in aromatic amino acids, termed the caveolin-binding domain, and mutations in this domain abrogate sterol binding both *in vivo* and *in vitro* (Choudhary et al., 2014; Darwiche et al., 2017a). To test whether the CAP domain of Pry3 also binds sterols and whether this sterol binding function is important for the observed mating phenotype, we expressed hexahistidine-tagged versions of the CAP domains of Pry1 and Pry3 in *Escherichia coli* and purified the proteins by affinity chromatography. *In vitro* binding assays with radiolabeled [^3^H]cholesterol revealed that the CAP domain of Pry3 exhibits saturable sterol binding with a *K_d_* of 13.08 µM (Fig. 3B). Exchange of a crucial cysteine residue, known to be engaged in a disulfide bridge, by serine in the C-terminal part of the CAP domain of Pry1, Pry1^C279S^, has previously been shown to abrogate both *in vivo* sterol export and *in vitro* sterol binding (Choudhary et al., 2014). Introduction of this mutation into Pry3, as present in Pry3-CAP^C142S^, disrupted sterol binding *in vitro*. In addition, sterol binding by the CAP domain of Pry3 was dependent on the flexibility of the loop region containing the caveolin-binding motif, because mutations designed to clamp that loop region by the introduction of a disulfide bridge between proline 105 and alanine 155, decreased sterol-binding of Pry3-CAP^P105C, A155C^ to background levels, i.e. levels of [^3^H]cholesterol observed in the absence of added protein (Fig. 3B).

To test whether sterol-binding by Pry3 is important for the mating inhibition we generated full-length versions of Pry3 in which cysteine 142 in the C-terminal part of the CAP domain was exchanged to serine, Pry3^C142S^, and a version of Pry3 with a clamped-down caveolin-binding loop, Pry3^P105C, A155C^. Expression of Pry3^C142S^ abrogated the mating inhibition, whereas expression of the clamped version Pry3^P105C, A155C^ still conferred mating inhibition (Fig. 3C). Given the fact that the Pry3 double mutant version, Pry3^P105C, A155C^, did not bind sterols *in vitro* but still conferred mating inhibition, we conclude that sterol binding is not required for inhibiting mating. The observation that the cysteine 142 mutant of Pry3 did not inhibit the mating reaction may be explained by the possibility that a lack of the disulfide bridge in the C-terminal part of the CAP domain may affect proper folding, stability and/or localization of the protein (see below) (Szyperski et al., 1998). This proposition is consistent with the fact that the cysteine 142 mutant is behaving like a loss-of-function mutation as it affects both sterol binding and mating inhibition. Taken together, these results indicate that sterol-binding by Pry3 is not required for its function in mating inhibition.

### Palmitate-binding is not essential for mating inhibition by Pry3

Tablysin-15, a CAP superfamily member from the horsefly *T. yao* has previously been shown to bind eicosanoids as well as free fatty acids in a hydrophobic pocket formed by helices α1 and α3 of the CAP domain (Xu et al., 2012). Fatty acid binding is conserved in yeast Pry1 and is physiologically important for cells to survive an excess of intracellular free fatty acids (Darwiche et al., 2017b). To test whether Pry3 can bind fatty acids, we performed *in vitro* binding studies using [^3^H]palmitic acid as radioligand. The purified CAP domain of Pry3 bound the radioligand with saturable kinetics and a *K_d_* of 114 µM (Fig. 3D). The affinity of Pry3 to palmitic acid is thus similar to that of Pry1 (*K_d_* of 111 µM). To determine whether fatty acid binding by Pry3 is important for its function in mating inhibition, we generated a mutant version in which valine at position 117 of Pry3 is exchanged for methionine. The corresponding valine 254 in Pry1 points into the fatty acid-binding pocket and its substitution by methionine is known to affect fatty acid binding of Pry1 (Darwiche et al., 2016, 2017b). Pry3^V117M^ failed to efficiently bind palmitic acid *in vitro* as the resulting binding curve was close to background binding (Fig. 3D).

To test whether fatty acid-binding is required for the function of Pry3 in mating inhibition we generated a full-length version of the valine 117 mutant of Pry3. When tested in the quantitative mating assay, overexpression of Pry3^V117M^ resulted in mating inhibition comparable to wild-type Pry3, indicating that fatty acid-binding is not essential for the inhibitory function of Pry3 in the mating process (Fig. 3C). Thus, the results obtained so far indicate that the function of Pry3 in the yeast mating process is independent of the lipid-binding properties of the CAP domain.

### Highly conserved residues within the CAP domain are required for inhibition of mating by Pry3

Structural analysis of a plant PR-1 (pathogenesis related 1) protein and its comparison to human glioma pathogenesis-related protein (GliPR) led to the identification of a common partially solvent-exposed spatial cluster of four amino acids: histidine 69, glutamic acid 88, glutamic acid 110, and histidine 127 (in the GliPR nomenclature). The conservation of this cluster indicates a common putative active site and molecular modeling suggested that these residues could adopt a conformation as found in the active site histidyl- and glutamyl-residues of several Zn^2+^-dependent proteases (Szyperski et al., 1998).

**Fig. 4.**
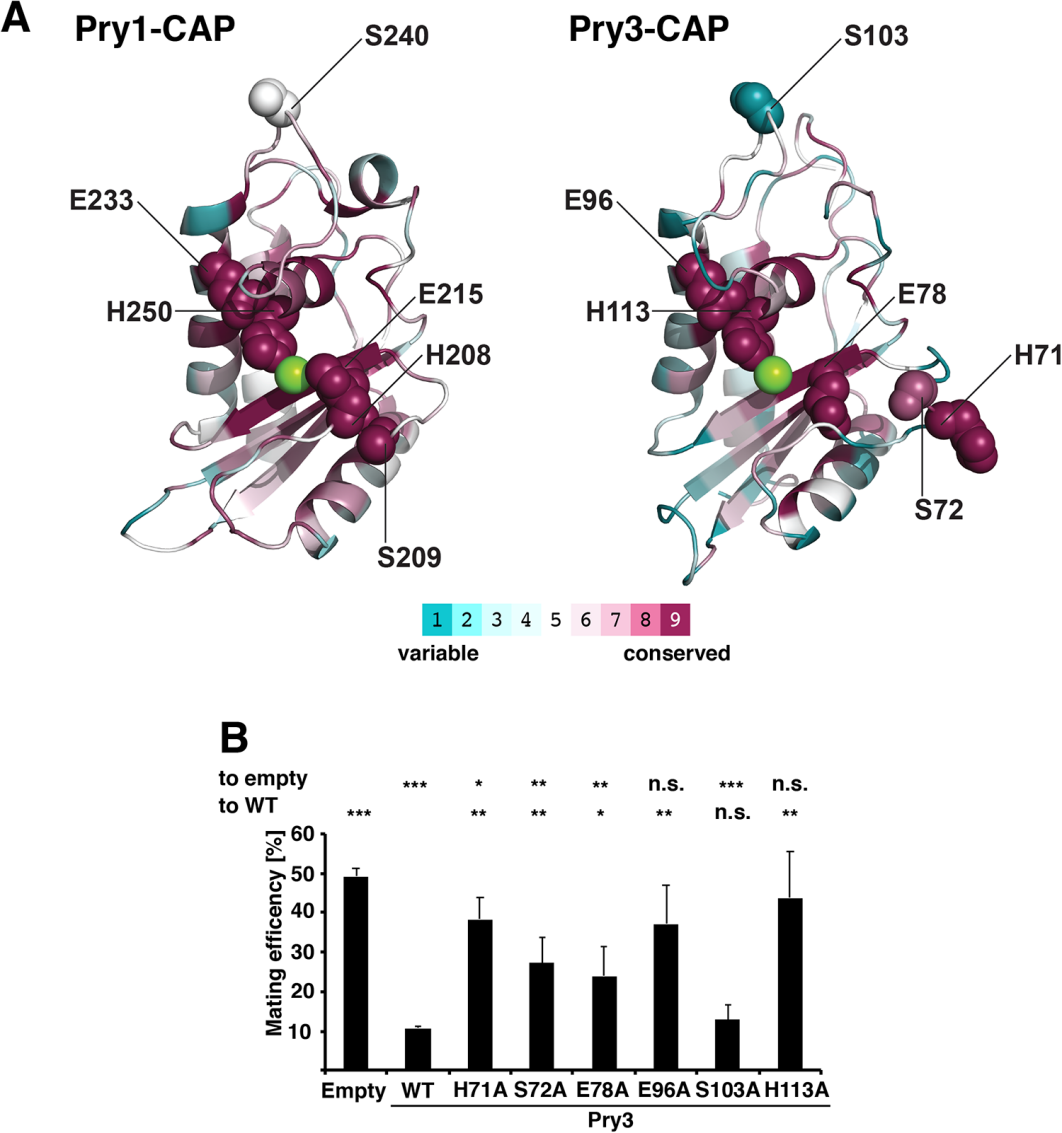
**Mating inhibition by Pry3 requires putative active site residues.** (A) Analysis of conserved surface residues of the CAP domain of Pry1 and Pry3. A ConSurf analysis was performed on the CAP domain of Pry1 and Pry3. Purple indicates highly conserved amino acids, whereas variable residues are in cyan as depicted in the color code bar. The central cation is indicated in green. The putative active site residues histidine 71, serine 72, glutamic acid 78, glutamic acid 96 and histidine 113, which line the central CAP cavity are highly conserved, while serine 103, which is located far away from the proposed active site shows only low conservation. (B) Conserved putative active site residues of Pry3 are required for mating inhibition. Cells containing an empty plasmid, a plasmid overexpressing wild-type Pry3, or the indicated mutant forms of Pry3 were mated to wild-type cells and mating efficiency was quantified. Values represent means±s.d. of four independent experiments. Asterisks denote statistical significance with respect to cells containing the empty plasmid or to cells overexpressing wild-type Pry3 (WT) (Welch *t*-test; *P-value <0.05; **P-value <0.01; ***P-value<0.001; n.s., not significant).

Interestingly, analysis of evolutionary conservation between the CAP domains of Pry1 and Pry3 using ConSurf, revealed that the four amino acids mentioned above: histidine 71, glutamic acid 78, glutamic acid 96 and histidine 113 (in the Pry3 nomenclature) showed the highest degree of conservation (Ashkenazy et al., 2016) (Fig. 4A). These residues are lining a large central groove, known as the CAP cavity, suggesting that these slowly evolving residues are crucial for CAP domain function.

In support of a possible catalytic function of the CAP domain, two of these putative active site residues, glutamic acid 96, and histidine 113 (in Pry3 nomenclature), have been postulated to be important for the protease function of the cone snail CAP family member, Tex31 (Milne et al., 2003). In Tex31, these evolutionary conserved residues could form part of a catalytic triad together with serine 80 as the catalytic nucleophile (corresponding to serine 72 in Pry3) (Fig. 4A).

To test whether these highly conserved residues are important for the function of the Pry3 CAP domain in mating inhibition, we generated the respective point mutant versions and tested them in the mating assay. Mutations in the presumed active site and/or ion-binding residues, histidine 71, serine 72, glutamic acid 78, glutamic acid 96 and histidine 113 resulted in significant attenuation of mating inhibition (Fig. 4B). Mutation of a peripheral, non-active site residue, serine 103, on the other hand, did not significantly affect mating efficiency. These putative active site mutations are unlikely to affect the folding and/or stability of the protein as similar mutations in Pry1 have previously been shown to retain the sterol-binding function of Pry1 and simultaneous exchange of some of these residues lining the CAP cavity in GAPR-1 abrogated binding to Beclin-1 but still retained the characteristic fold of the CAP domain (Choudhary et al., 2014; Li et al., 2017). These residues are thus likely to affect a possible catalytic, maybe proteolytic function of the CAP domain. This is in line with studies on Fpr1, a PR-1 like CAP protein from *Fusarium oxysporum* that functions in virulence on mammalian but not plant hosts. Fpr1, encodes a predicted GPI-anchored CAP protein that is proteolytically processed by the fungus. Fpr1 is required for virulence in a disseminated immunodepressed mouse model, and like Pry3, its function depends on the integrity of these proposed active site residues (Prados-Rosales et al., 2012).

### Pry3 displays polarized cell surface localization

**Fig. 5.**
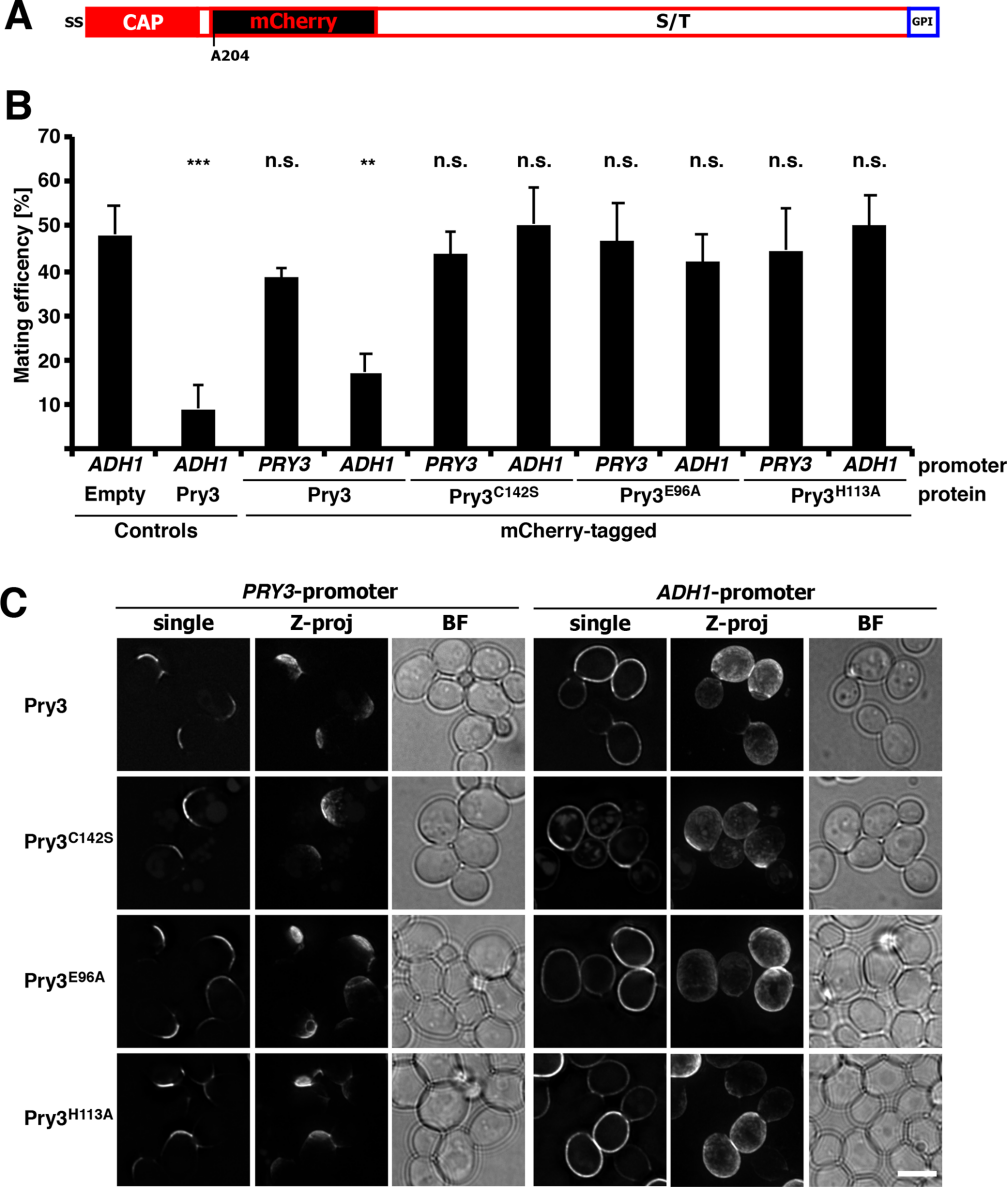
**Pry3 displays polarized localization at the cell surface.** (A) Structure of an internally mCherry-tagged version of Pry3. The mCherry open reading frame was inserted after the CAP domain of Pry3, after alanine 204. (B) The mCherry-tagged versions of Pry3 is functional in mating inhibition. Mating inhibition of the indicated mCherry-tagged versions of Pry3 expressed either from a strong *ADH1* promoter or from the endogenous *PRY3* promoter was assessed using the quantitative mating assay. Values represent means±s.d. of four independent determinations. Asterisks denote statistical significance (Welch *t*-test; **P-value <0.01; ***P-value <0.001; n.s., not significant). (C) Polarized surface localization of Pry3. Plasmid-borne copies of wild-type and the indicated mutant versions of mCherry-tagged Pry3 were analyzed by fluorescent microscopy in living cells. Pry3 was expressed either from its endogenous promoter or from an *ADH1*-promoter. When expressed from the endogenous promoter, Pry3 displays polarized cell surface localization whereas if overexpressed from the *ADH1*-promoter, Pry3 shows uniform cell surface localization. Shown are deconvolved images of either a single optical section through the middle of the cell (single), or a Z-projection (Z-proj). Scale bar: 5 µm; BF, bright field illumination.

To further understand how overexpression of Pry3 results in mating inhibition, we decided to analyze the subcellular localization of the protein. Therefore, we first generated a fluorescent mCherry-tagged version of the protein. Since the N-terminus of Pry3 contains a signal sequence and the C-terminus harbors the omega-site required for GPI-anchoring, we tagged the protein internally, after the CAP domain but before the serine/threonine rich region (Fig. 5A). Insertion of the mCherry open reading frame flanked by 4×(glycine-alanine) linker after alanine 204 preserved the functionality of the protein in mating inhibition (Fig. 5B). Mating inhibition, however, was dependent on overexpression from an *ADH1* (alcohol dehydrogenase 1) promoter, since expression of the mCherry-tagged version of Pry3 from its endogenous *PRY3* promoter did not affect mating efficiency.

Localization of this mCherry-tagged version of Pry3 by fluorescence microscopy revealed polarized crescent-like localization of the protein at the cell periphery, most likely the cell wall (Fig. 5C). When the mCherry-tagged version of Pry3 was overexpressed from the *ADH1* promoter, the polarized distribution, observed with the protein expressed from its native promoter, was replaced by a more uniform cell surface localization. This altered localization indicates that overexpression of Pry3 results in a loss of its polarized distribution, and suggests that the observed mating inhibition may be due to mislocalization of the protein to surface domains, where it may exert an inhibitory function on the mating process.

To examine whether the putative active site mutant versions of Pry3, Pry3^E96A^ and Pry3^H113A^, and the disulfide bridge mutant, Pry3^C142S^, are all properly expressed and localized to the cell surface, we generated mCherry-tagged versions of these alleles. When expressed from the endogenous promoter all three mutant proteins displayed polarized crescent-like surface localization. When overexpressed from an *ADH1* promoter, Pry3^E96A^ and Pry3^H113A^ displayed a more uniform distribution over the cell surface comparable to that of overexpressed wild-type Pry3. These results indicate that the loss of mating inhibition of these point mutant alleles is not due to their decreased expression or rapid turnover, but likely due to a loss-of-function of the protein itself. On the other hand, overexpression of the disulfide bridge mutant allele, Pry3^C142S^, resulted in a somewhat more polar localization than was observed with either the wild type or the Pry3^E96A^ and Pry3^H113A^ mutant versions (Fig. 5C). Lack of mating inhibition of Pry3^C142S^ should thus be interpreted cautiously, since it could be due to either a loss-of-function of the protein, a reduction of expression levels, or a non-uniform cell surface localization.

**Fig. 6.**
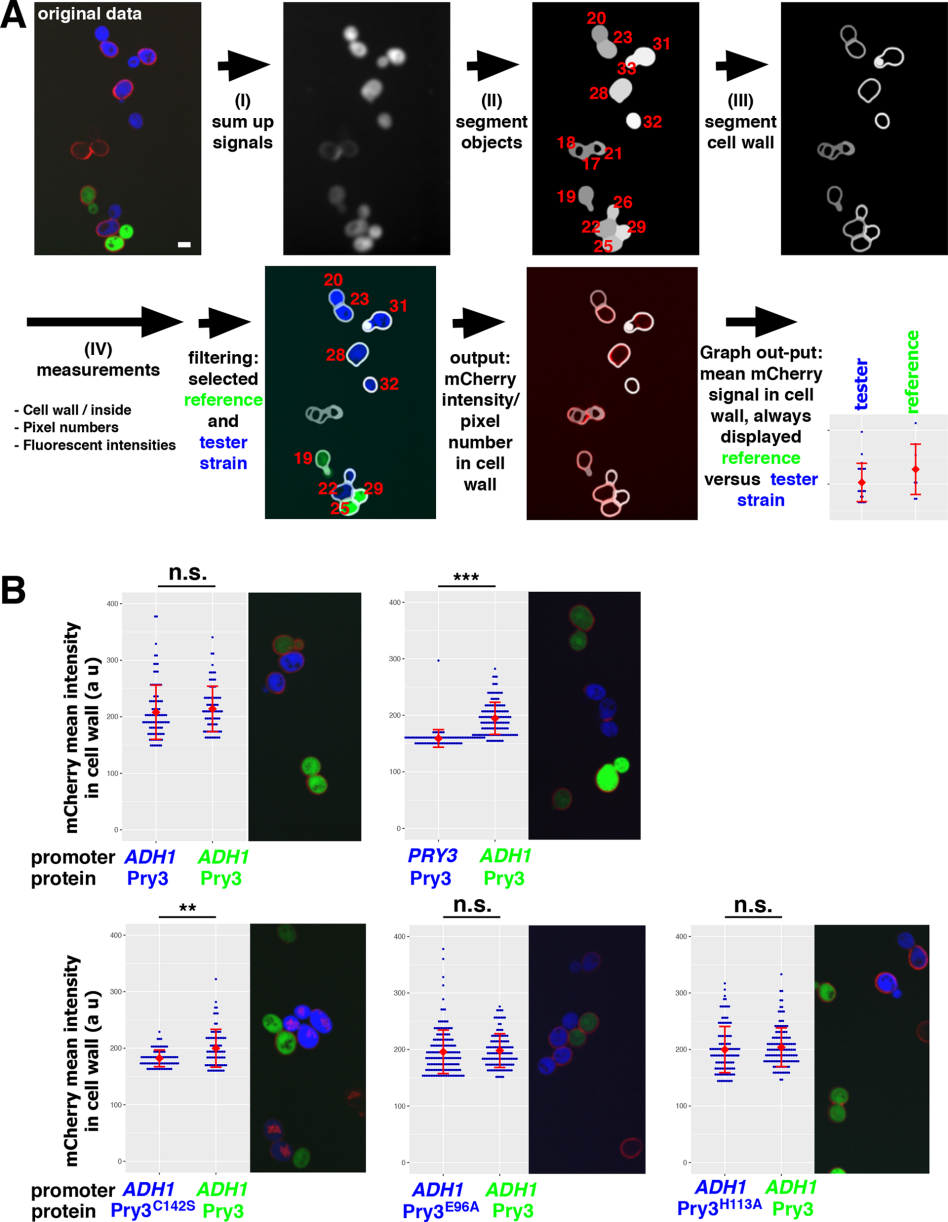
**Quantification of Pry3-mCherry fluorescence in the cell wall.** (A) Workflow to quantify fluorescence intensity of mCherry fusion proteins in the cell wall. Macros were applied to original raw data to determine fluorescence intensities within the cell and in cell wall as segmented. The identity of the cells as either reference strain, containing cytosolic Citrin (green), or tester strain, expressing cytosolic CFP (blue), and mean mCherry intensity in the cell wall were determined and compared in a dot plot. (B) Cell surface levels of the E96A mutant version of Pry3 are similar to that of wild-type Pry3. Comparison of the mean intensity of genomically expressed Pry3-mCherry fusion proteins in the cell wall between a reference strain (*ADH1-PRY3-mCherry*) and different tester strains. Comparison between the reference strain and itself and a strain expressing Pry3-mCherry from its endogenous *PRY3* promoter. Single optical sections are shown. Scale bar: 5 µm. Blue dots, mean mCherry intensity in cell wall of a single cell; red, mean of the mean±s.d.; *n*>80; statistics, Mann–Whitney *U*-test; **P-value <0.01; ***P-value <0.001; n.s., not significant.

Since western blotting could not be used to assess the expression levels of the mCherry-tagged mutant Pry3 proteins, we developed macros to measure cell wall associated Pry3-mCherry fluorescence in tester strains expressing cytosolic CFP relative to a reference strain expressing cytosolic Citrin within the same field of view and hence identical exposure settings (see Materials and Methods; Fig. 6A). This analysis clearly allowed to distinguish between the fluorescence levels of cells expressing genomically mCherry-tagged Pry3 under the control of the endogenous promoter from those overexpressing it from a strong *ADH1* promoter (Fig. 6B). Genomic tagging and expression of Pry3 in these experiments was important to obtain low variations of expression levels between cells within a population. Comparison of cells expressing mutant versions of Pry3 revealed that the putative active site mutants E96A and H113A were expressed at levels similar to that of wild-type Pry3 (Fig. 6B). The disulfide bridge mutant variants, C142S, on the other hand were expressed at lower levels than wild-type Pry3. Taken together, these results indicate that the lack of mating inhibition of the putative active site mutant versions of Pry3, E96A and H113A, are not due to mislocalization or reduced levels of expression of these proteins, but likely due to a genuine loss-of-function of CAP domain in a process affecting mating inhibition.

### Endogenous Pry3 is absent from mating projections

**Fig. 7.**
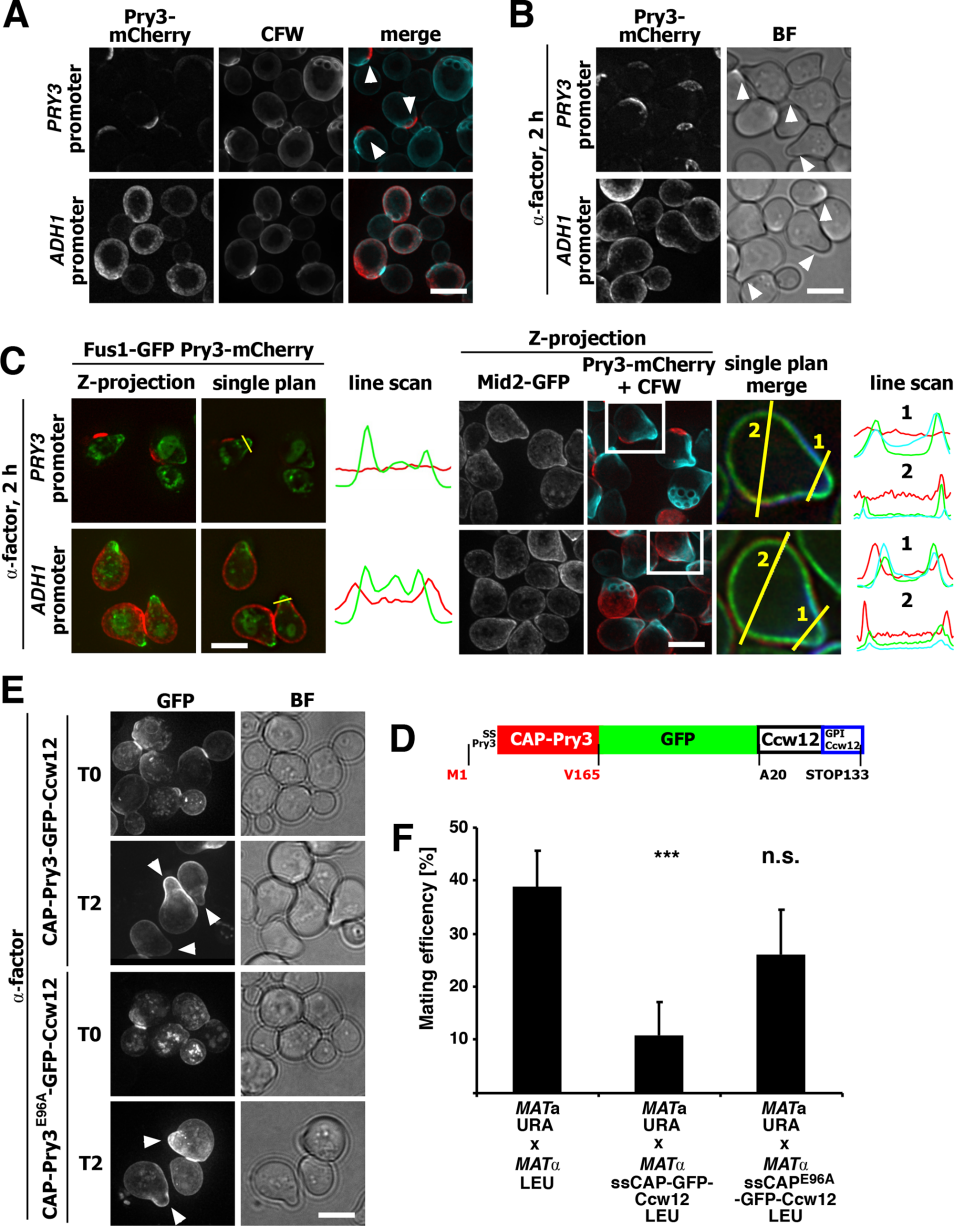
**Endogenous Pry3 is absent from mating projections.** (A) Pry3 localizes adjacent to bud scars. Bud scars were stained with calcofluor-white (CFW) and colocalized with mCherry-tagged Pry3 expressed from its endogenous promoter or from the *ADH1* promoter. Arrowheads mark the crescent-like localization of Pry3-mCherry. (B) Pry3 localizes next to mating projections. Cells expressing mCherry-tagged Pry3 either from its endogenous promoter or from the *ADH1*-promoter were treated with alpha-factor for 2 h. Arrowheads point to mating projections. (C) Overexpression of Pry3 results in its mislocalization onto mating projections marked by Fus1-GFP, Mid-GFP, or CFW. Cells coexpressing Fus1-GFP or Mid2-GFP with mCherry-tagged Pry3, either from its endogenous promoter or from an *ADH1* promoter, were treated with mating factor for 2 h prior to imaging. Line scans indicate the presence of Pry3-mCherry at the neck of mating projections in cells overexpressing Pry3. (D) Schematic representation of the fusion construct between the CAP domain of Pry3, GFP and the GPI-anchored cell wall protein Ccw12. The CAP domain of Pry3 is represented by the red bar, GFP by the green bar and Ccw12 by the white bar. Sites of fusions are indicated by amino acid positions. (E) The CAP-Ccw12 fusion protein localizes to mating projections. Cells expressing the wild-type or E96A mutant version of the fusion protein were treated with alpha-factor and their localization was analyzed by fluorescence microscopy. Arrowheads mark mating projections. Scale bars: 5 µm; BF, bright field illumination. (F) The CAP-Ccw12 fusion protein reduces mating efficiency. Wild-type cells were mated to cells overexpressing the fusion with a wild-type CAP domain (CAP-Ccw12) or the E96A mutant version of the CAP domain (CAP^E96A^-Ccw12) and mating efficiency was quantified. Values represent means±s.d. of two independent experiments. Asterisks denote statistical significance (Welch *t*-test; ***P-value <0.001; n.s., not significant).

To examine the nature of the crescent-like surface localization of Pry3, we examined whether Pry3 colocalizes with bud scars. Therefore, cells expressing Pry3-mCherry were stained with the fluorescent dye calcofluor-white, which specifically stains these bud scars. Pry3-mCherry surface crescents were consistently localized adjacent the calcofluor-white stained bud scars, indicating that Pry3 is localized to highly polarized regions of the cell surface (Fig. 7A).

Given that overexpression of Pry3 results in uniform cell surface localization and the possibility that this ectopic localization may be responsible for the observed mating inhibition, we wondered whether Pry3 may be present on mating projections (shmoos). Mating projections represent polarized regions of the cell surface formed by haploid cells that sense the presence of the mating pheromone of the opposite mating type (Merlini et al., 2013). To examine the localization of Pry3 in shmooing cells, *MAT**a*** cells expressing genomically mCherry-tagged Pry3 were treated with alpha mating factor for 2 h prior to imaging. When expressed from its endogenous promoter Pry3-mCherry displayed crescent-like polarized surface distribution but the protein was absent from mating projections (Fig. 7B). In overexpressing cells, the protein displayed a more uniform surface distribution, is was clearly present on the shmoo but hardly detectable on the shmoo tip (Fig. 7B).

To examine a possible localization of Pry3 on the mating projections more thoroughly, we performed colocalization experiments with proteins that specifically mark mating projections such as Mid2, a sensor of the cell wall integrity pathway (Philip and Levin, 2001), or Fus1, a plasma membrane anchored protein required for efficient cell–cell fusion during conjugation (Trueheart and Fink, 1989) (Fig. 7C). In cells expressing Pry3 under the *ADH1* promoter, Pry3 partially overlapped with Fus1 or Mid2 in the neck of the mating projection (Fig. 7C). A similar picture emerged from analyzing shmooing cells stained with calcofluor-white (Fig. 7C). In these cells, mCherry-tagged Pry3 was localized to a crescent-shaped surface domain next to bud scars, but not on the shmoo, when expressed from its endogenous promoter. When overexpressed from the *ADH1* promoter, however, the protein localized over the entire cell surface, including the shmoo neck (Fig. 7C).

To test whether the inhibitory action of Pry3 on mating is due to its leakage onto mating projections, we fused the CAP domain of Pry3 to Ccw12, a GPI-anchored cell wall protein that is present on shmoos (Ragni et al., 2011) (Fig. 7D). This fusion protein is present at the cell surface, but also the bud neck and in intracellular punctate structures (Fig. 7E). In cells treated with alpha-factor, the fusion protein localizes to mating projections. Consistent with the proposition that the presence of the Pry3 CAP domain on mating projections is mating inhibitory, cells expressing the Ccw12-based fusion protein displayed reduced mating efficiency (Fig. 7F). This reduction in mating efficiency was due to the presence of a functional CAP domain as fusions expressing the mutant CAP domain, CAP^E96A^, did not affect mating efficiency (Fig. 7F), despite the fact that the fusion protein was localized to mating projections (Fig. 7E).

### A surface proximal CAP domain displays dose-dependent inhibition of mating

**Fig. 8.**
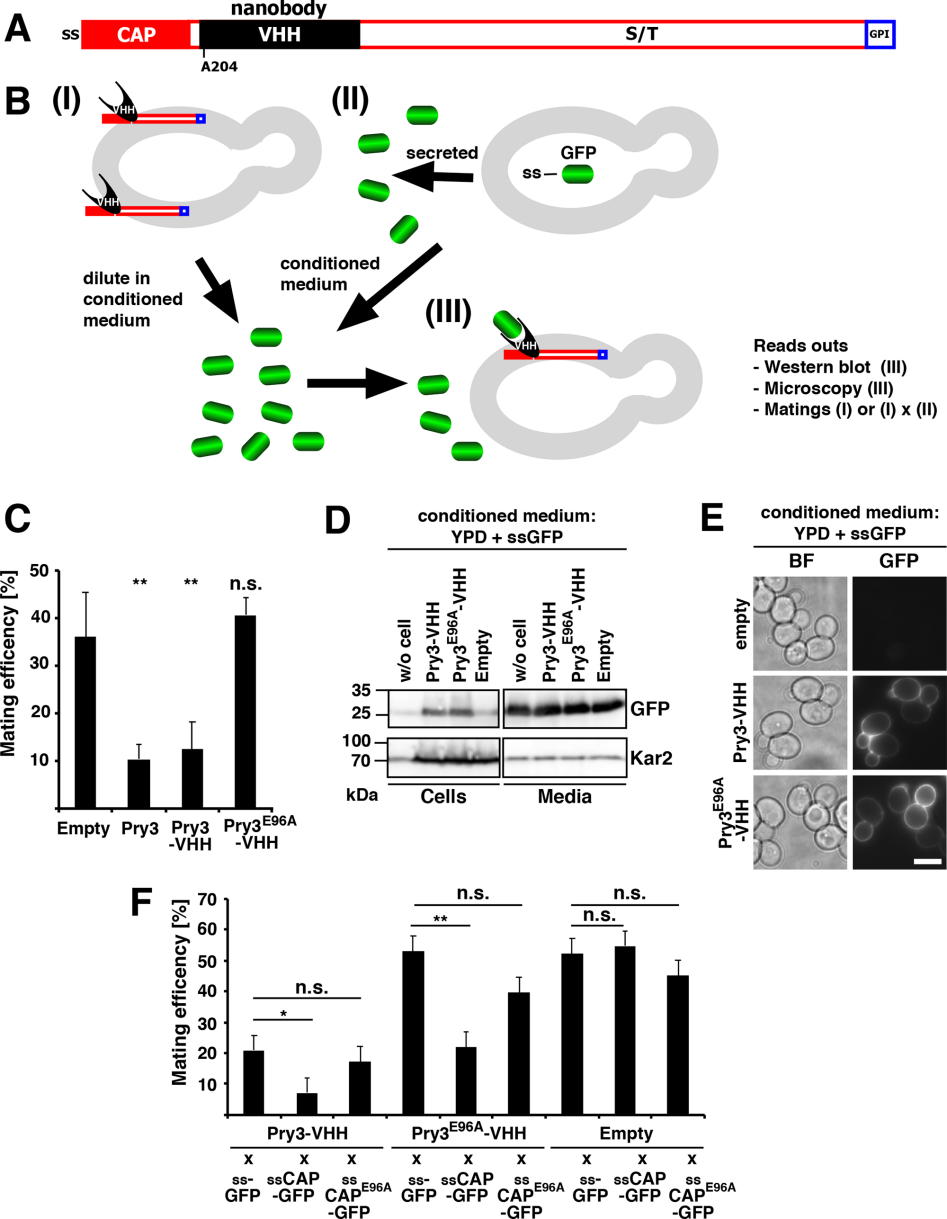
**Cell wall association of the CAP domain results in a dose-dependent mating inhibition.** (A) Schematic representation of cell-wall attached Pry3 internally tagged with the single-domain antibody VHH, which binds GFP. Pry3 is represented by a close red box for the CAP domain, a black box for the VHH internal tag, an open red box for the serine/threonine rich region, and a blue box for the GPI-anchor site. (B) Schematic representation of the nanobody-based assay system. (I) The nanobody-containing fusion protein is localized to the yeast cell wall (grey oval), exposed to the culture medium and thus can interact with GFP (green barrel) in the medium. (II) Cultivation of cell expressing a signal sequence (ss) containing GFP results in the secretion of ss-GFP into the culture medium. Tester strains (I) are then diluted in the conditional medium containing ss-GFP (III) and assayed for levels of the nanobody containing fusion protein by western blotting, its localization and functionality. (C) VHH-tagged Pry3 is functional in mating inhibition. Mating efficiency of cells containing an empty plasmid or overexpressing wild-type Pry3, Pry3-VHH, or Pry3^E96A^-VHH. Statistical analysis was performed as detailed in panel F. (D) Pry3-VHH and Pry3E96A-VHH are present at similar levels. Proteins from cells expressing Pry3-VHH, Pry3^E96A^-VHH or an empty vector as well as the conditioned culture medium containing ssGFP were analyzed by western blotting using an anti-GFP antibody. Equal signal intensity of GFP present in the conditioned medium used to cultivate cells expressing the different plasmids indicates that the availability of free GFP for binding to VHH is not limiting. Kar2 detection serves as loading control. (E) Pry3-VHH and Pry3^E96A^-VHH are localized to the yeast cell wall and bind GFP present in the conditioned medium. Cells containing an empty vector or expressing Pry3-VHH and Pry3^E96A^-VHH under the control of *ADH1* promoter were cultivated in conditioned YPD medium containing free GFP for 4 h and analyzed by epifluorescence microscopy. BF, bright field; scale bar: 5 µm. (F) The CAP domain inhibits mating in a dose-dependent manner when localized to the cell wall. Quantitative mating assay of cells expressing the nanobody-containing wild-type Pry3, the E96A mutant version, or an empty plasmid. Cells were mated with strains expressing the secreted GFP (ss-GFP), secreted CAP domain (ss-CAP-GFP), or a E96A mutant version of the secreted CAP domain (ss-CAP^E96A^-GFP). (Welch *t*-test; *P-value <0.05; **P-value <0.01; n.s., not significant).

To validate the importance of the putative active site residues within the CAP domain on mating inhibition we generated a variant of Pry3 in which a GFP-binding Camel single-domain antibody fragment (VHH, nanobody) was inserted after the CAP domain (Rothbauer et al., 2006; Saerens et al., 2005) (Fig. 8A). A wild-type Pry3-VHH fusion was functional in mating inhibition, however, a mutant Pry3^E96A^-VHH version was non-functional in mating inhibition, consistent with the importance of the conserved glutamic acid at position 96 of Pry3 (Fig. 8C). GFP-binding through the VHH domain present in both wild-type and the E96A point mutant version of Pry3 allowed us to (i) compare protein abundance by western blotting for bound GFP, which is not quantifiable for full length Pry3 (Fig. 1C), and (ii) to visualize the localization of the protein by fluorescence microscopy. To compare protein abundance of wild-type Pry3 to that of the E96A mutant, we cultivated cells expressing the VHH-fusion proteins in conditioned medium containing free GFP (Fig. 8B). This conditioned medium was generated by first cultivating cells expressing a secreted version of GFP (ssGFP) and the medium was then used to grow cells expressing the Pry3-VHH fusions. Under these conditions, the Pry3-VHH fusion quantitatively bound the free GFP from the conditioned medium, thus allowing us to ascertain that the E96A mutant version of Pry3 was synthesized and transported to the cell surface at levels comparable to that of wild-type Pry3 (Fig. 8D). When analyzed by fluorescence microscopy, cells expressing these VHH-fusions displayed GFP fluorescence at the cell perimeter, consistent with a cell wall localization of Pry3 (Fig. 8E).

Finally, we tested whether, a CAP domain that is trapped in the cell wall and hence in proximity to the plasma membrane would be sufficient to inhibit the mating reaction. Therefore, we generated cells secreting a GFP-tagged CAP domain (ss-CAP-GFP). These cells were then mated with cells expressing the nanobody-tagged Pry3 (Pry3-VHH) which is expected to bind ss-CAP-GFP and thereby increase the relative abundance of CAP domains in proximity of the plasma membrane of the mating partner. When tested with the quantitative mating assay, cells expressing ss-CAP-GFP displayed a significantly reduced mating efficiency compared to cells expressing only ss-GFP, indicating that an increase in abundance of the CAP domain in the cell wall is sufficient to reduce mating efficiency (Fig. 8F). Expression of a soluble ss-CAP-GFP alone, however, did not impact mating as mating efficiency was not reduced when these cells were mated with cells that did not express a nanobody tagged Pry3 (Fig. 8F). Similarly, expression of the E96A mutant version of the CAP domain did not reduce mating efficiency regardless of whether it was expressed as the GPI-anchored nanobody fusion or the secreted CAP domain. However, the mating efficiency of cells expressing the E96A mutant version of the nanobody-containing Pry3 was complemented by cells expressing the wild-type version of the secreted CAP domain, but not the mutant CAP domain, confirming that the CAP domain is sufficient for mating inhibition when present in the cell wall (Fig. 8F).

Taken together, the data presented here indicate that mating inhibition by Pry3 is a conserved function of the CAP domain that is independent of its ability to bind lipids. Mating inhibition, however, depends on cell wall association of the CAP domain and on highly conserved residues possibly forming a putative catalytic active site in the CAP domain. Pry3 is localized to a highly polarized crescent-shaped cell wall domain adjacent to bud scars. Overexpression, which is required for mating inhibition, results in uniform surface localization of the protein and hence in mislocalization of the protein on mating projections. The presence of Pry3 on these mating projections is likely required for mating inhibition as illustrated by the mating inhibition of cells expressing the fusion of the CAP domain with Ccw12. What process the presence of the CAP domain on mating projections precisely affects, is presently unknown. However, it is interesting to note that CAP family proteins have been strongly implicated in cell–cell fusion processes in other organisms, because they affect fertilization in mammals, amphibians and invertebrates such sea squirts (ascidians) (Burnett et al., 2012; Gibbs et al., 2008; Urayama et al., 2008). CRISP1, for example, the first identified member of the highly evolutionary conserved cysteine-rich secretory protein (CRISP) family, modulates mammalian sperm motility and orientation during fertilization (Ernesto et al., 2015). CRISP members are characterized by the presence of a cysteine-rich domain (CRD, also known as ICR, ion channel regulatory domain), which is connected through a hinge region to the CAP domain (Abraham and Chandler, 2017). While the CRD, possibly in combination with the hinge region, has been implicated in inhibiting Ca^2+^ channel activity, the precise molecular function of the CAP domain in fertilization remains elusive. Employing the yeast-based mating assay described in this study, we hope to further define the mode of action of CAP family members in this still poorly understood cell–cell fusion process.

## MATERIALS AND METHODS

### Yeast strains, growth conditions, epitope tagging and site-directed mutagenesis

Yeast mutant strains were cultivated either in rich media, YPD (containing 1% Bacto yeast extract, 2% Bacto peptone, and 2% glucose, US Biological Swampscott, MA, USA), or in minimal defined media (containing 0.67% yeast nitrogen base without amino acids (US Biological), 0.73 g/l amino acids, and 2% glucose). Yeast strains used in this study and their genotype are listed in Table S1.

To overexpress Pry1, Pry2 and Pry3, their coding sequence was amplified from genomic DNA and recombined into the high-copy number plasmid pRS426-*ADH1^prom^* cut with XmaI (for plasmids see Table S2). To exchange the selection maker, *LEU2* coding sequence was amplified by PCR and recombined into pRS426-*ADH1^prom^-PRY3* linearized with NcoI and PstI restriction enzymes (cut within *URA3*). To obtain HA-tagged version of Pry1 and Pry2, the overexpression plasmids (p1314, p1311) were digested with XhoI and SalI, respectively, and then re-circularized with primers containing recombination arms recognizing the 3′-end of the genes as well as the sequence encoding for the HA tag (YPYDVPDYA). Pry3 was tagged internally, after alanine 204, with HA tag using a PCR-ligation-PCR approach (Ali and Steinkasserer, 1995). The PCR product was recombined into Pry3 overexpression plasmid digested with SpeI.

To facilitate the production of various Pry3-based plasmids, we introduced a unique XmaI restriction site either after the signal sequence (proline 23), or following the CAP domain (alanine 204) by a PCR-ligation-PCR approach (Ali and Steinkasserer, 1995), followed by recombination into pRS426-*ADH1^prom^* (Christianson et al., 1992) to produce *ADH1^prom^-Pry3-ssXmaI* and *ADH1^prom^-Pry3-AAXmaI*, respectively. Truncated variants of *PRY3* were generated by amplifying fragments of *PRY3* using primers containing premature stop codons or alternative start codons, and amplified fragments were inserted into pRS426-*ADH1^prom^*, linearized with XmaI, by *in vivo* recombination. Introducing a stop codon after glycine 853 yielded Pry3-ΔGPI, a stop after isoleucine 162 yielded Pry3-CAP, using methionine 190 as a start codon gave Pry3-ST. For the truncation of the S/T rich region (Pry3-ΔST), again the PCR-ligation-PCR strategy was applied and resulted in a plasmid expressing Pry3 missing amino acids alanine 192 to glutamic acid 840. For the deletion of the CAP domain (Pry3-ΔCAP), custom primers were designed to remove the sequence encoding asparagine 24 to isoleucine 162, and replacing it by glycine-alanine-glycine. The primers were annealed together and recombined into *ADH1^prom^-PRY3-ssXmaI* linearized with XmaI.

To exchange the CAP domain of *PRY3* with that of *PRY1* and *PRY2*, the sequence of *PRY1* encoding amino acids serine 160 to leucine 298 and that of *PRY2* encoding the CAP domain from serine 190 to leucine 328 were amplified with primers that allowed *in vivo* recombination mediating deletion of the CAP domain of *PRY3* (amino acid asparagine 24 to leucine 161) in XmaI linearized *ADH1^prom^-PRY3-ssXmaI*.

For site-directed mutagenesis, *ADH1^prom^-PRY3-ssXmaI* was cut with XmaI and BamHI and PCR products carrying site-directed mutations were inserted by homologous recombination.

Pry3 was internally tagged by adding the single domain antibody VHH flanked by 4x(glycine-alanine) linkers on both sides and inserted after alanine 204 to generate Pry3-VHH. VHH was amplified from plasmid pRH2776 (Powis et al., 2015) and recombined into *ADH1^prom^-Pry3-AAXmaI*, linearized with XmaI*.* A tagged E96A mutant version of Pry3 was obtained using the same strategy as for the untagged mutant, but using Pry3-VHH as template for the PCR-ligation-PCR. For internal mCherry-tagging of Pry3, mCherry was amplified from plasmid pBS34 (Hailey et al., 2002) and recombined into *PRY3* as described for VHH-tagging. To generate the mCherry tagged mutant versions of Pry3, Pry3-AAmCherry was used as template to insert the point-mutations. To allow expression of mCherry-tagged versions of Pry3, under its endogenous promoter, we first inserted Pry3 coding sequence flanked by 664 bases upstream of the start codon and 523 bases downstream of the stop codon into the centromeric vector pGREG506 (Jansen et al., 2005), cut with SalI and SacI, yielding *PRY3^prom^-PRY3*. This plasmid was then digested with SpeI to introduce mCherry, and with AscI and BamHI to introduce mCherry and the mutated CAP domains.

For the secretion of GFP into the culture medium, we amplified GFP from pKT128 (Sheff and Thorn, 2004) and *PGK1^prom^-KAR2^ss^* from p*KAR2^ss^-CFP-HDEL* (Szymanski et al., 2007), PCR-ligated them and recombined the product into pGREG503 linearized with SalI and AscI. *PGK1^prom^-PRY3^ss^-PRY3^CAP^-GFP* wild-type and E96A mutant were obtained by amplifying the CAP domains (amino acid methionine 1 to valine 165, with a reverse primer containing a 4x glycine-alanine linker) and introducing it after *PGK1^prom^* and before *GFP* by recombination in *PGK1^prom^- KAR2^ss^-GFP* digested with EcoRI and EcoRV.

Plasmids overexpressing the fusion protein Pry3^CAP^-GFP-Ccw12 wild type and E96A mutant were obtained by PCR-ligating Pry3^CAP^-GFP and Ccw12 (Fig. 7D) and recombination into pRS426 digested with XmaI. Leu2 selection marker was introduced as explained above.

All constructs were sequence verified (Microsynth AG, Buchs, Switzerland).

Epitope tagging of Hsp150 was performed by homologous recombination into the yeast genome of an amplified fusion cassette, pFA6a-13Myc-His3MX6 (Longtine et al., 1998). To genomically tag Pry3 with mCherry at position alanine-204, we first inserted a *URA3* cassette between serine 169 and threonine 242 of Pry3. This wild-type *MAT**a*** strain was then transformed with a DNA product obtained by cutting *PRY3^prom^-PRY3-mCherry* with AscI and SalI and transformants were counter selected on plates containing 5-fluoroorotic acid (Boeke et al., 1987). Next, we replaced the promoter of *PRY3* with that of *ADH1* to produce a strain overexpressing a genomic copy of *PRY3-mCherry*.

Point mutations in Pry3 were introduced into the genome of yeast strain RSY5886 with the help of the CRISPR/Cas9 system (Generoso et al., 2016). To do so, pRCC-K was amplified by PCR to introduce protospacers that detect DNA sequences closed to the desired mutations (for mutation H113A 3′-CAAATCCCGGATTTTCTGAATCCAC-5′, for E96A 3′-GAGCGGTGGACGCCTGGTACG-5′, and for C142S 3′-GTGTGCTCCTACAACCCTCC-5′), and the amplified fragments were circularized with the Gibson Assembly Cloning Kit (New England Biolabs, #E5510S). The plasmids were then transformed in yeast together with their respective DNA donors consisting of a single stranded oligonucleotide containing the desired mutations (for mutation H113A 3′-GTATAATTATTCAAATCCCGGATTTTCAGAGTCCACGGGTGCCTTCACACAGGTGGTTTGGAAGTCAACCGCCGAGATTG-5′, for E96A 3′-GTTACACAGACACGGGAGCGGTGGATGCATGGTACGGGGCGATAAGCAAGTATAATTATTCAAATCCCGGATTTTCTG-5′, and for C142S 3′-GTGGTACGACATGGAACAATTATATTGTGTCCTCCTACAATCCTCCTGGAAACTACCTGGGTGAGTTTGCAGAG-5′). Introduction of the mutations into yeast genome was controlled by amplifying the CAP domains and sequencing the PCR products.

Fus1-GFP and Mid2-GFP were obtained from the yeast GFP collection (Huh et al., 2003). Strains expressing two fluorescently labelled proteins were obtained by mating and sporulation.

### Protein secretion analysis and western blotting

The protocol was adapted from (Choudhary and Schneiter, 2012). Briefly, proteins were extracted from three OD_600_ units of yeast cells by NaOH followed by precipitation with 10% TCA. To analyze proteins in the culture supernatant, proteins from 20 ml media of an overnight grown culture were precipitated by 10% TCA.

The primary antibodies used were: anti-HA (rat, 1:2000, Roche #11867423001), c-Myc monoclonal antibody (mouse, 1:5000, Invitrogen #13-2500), monoclonal anti-GFP (mouse, 1:2000, Roche #11814460001), anti-Kar2 (rabbit, 1:5000, Randy Schekman, University of California at Berkeley, CA, USA). As secondary antibodies goat anti-rat IgG antibody, horseradish peroxidase (HRP) conjugate (1:10,000, Merck #AP136P), goat anti-rabbit IgG (H+L)-HRP conjugate (1:10,000, Bio-Rad #1706515) and goat anti-mouse IgG (H+L)-HRP conjugates (1:10,000, Bio-Rad #1706516) were employed.

### Quantitative mating assays

Quantitative mating assays were performed as described (Bickel and Morris, 2006). Briefly, 10^6^ cells of the experimental *MAT***a** strain (BY4741, containing *PRY3* on a *URA3* plasmid) were mixed with 10^7^ wild-type *MAT*α (BY4742) cells containing a *LEU2* plasmid. Cells were collected by vacuum filtration onto a 0.45 µm pore size nitrocellulose filter disk (Millipore, #N9020-100EA). Disks were incubated on top of YPD plates for 5 h at 30°C. Cells were resuspended in water, briefly sonicated, and diluted to titer the cells. Mating efficiency was calculated as the number of **a**/α diploids, growing on SD plates lacking both uracil and leucine, divided by the total number of colonies on SD plates lacking uracil (**a**/α diploids plus *MAT***a** haploids).

### Expression and purification of the CAP domain of Pry3

Wild-type and mutant versions of the CAP domain of Pry3, amino acids alanine 18 to lysine 161, were PCR amplified and cloned into the *NcoI* and *XhoI* sites of pET22b vector (Novagen, Merck, Darmstadt, Germany), which contains a *PelB* signal sequence to direct the secretion of the expressed protein into the periplasmic space. Plasmids were transformed into *E. coli* BL21 and the proteins were expressed as C-terminal polyhistidine-tagged fusions after lactose induction and overnight growth of the bacteria at 24°C. Cells were harvested, lysed and incubated with Ni-NTA beads (Qiagen, Hilden, Germany) as per the instructions of the manufacturer. Beads were washed and proteins were eluted with imidazole, and concentrated. Protein concentration was determined by Lowry assay using Folin reagent and BSA as standard.

### *In vitro* lipid binding assay

The radioligand binding assay was performed as described previously (Choudhary and Schneiter, 2012; Im et al., 2005). Purified protein (100 pmol) in binding buffer (20 mM Tris, pH 7.5, 30 mM NaCl, 0.05% Triton X-100) was incubated with [^3^H]cholesterol (100–500 pmol) or [^3^H] palmitic acid (100–500 pmol) for 1 h at 30°C. The protein was then separated from the unbound ligand by adsorption to Q-sepharose beads (GE healthcare, USA), beads were washed and the protein-bound radioligand was quantified by scintillation counting. To determine background binding, the binding assay was performed without addition of purified protein. *K_d_* values were calculated using Prism (GraphPad Prism, La Jolla, CA, USA).

### Fluorescence microscopy

For living yeast cell imaging, a DeltaVision Elite imaging system (GE Healthcare, Pittsburgh, PA, USA) was used. It consists of an Olympus 1X71 inverted microscope equipped with a CCD camera (CoolSNAP HQ2, Photometrics, Tucson, AZ, USA). Images were acquired with a U PLAN S-APO 100×1.42 NA oil immersion objective (Olympus). To image mCherry alone or together with GFP, a four-color fluorescent protein filter set optimized for GFP (excitation: 461–489 nm) and mCherry (excitation: 563–588 nm) visualization was used. Twenty-one to thirty-one 0.2 μm optical sections were deconvolved using the iterative constrained deconvolution program in softWoRx (Applied Precision). Single sections or maximal Z-projections are displayed in the figures. To image cells following calcofluor-white staining, the same set up was used except that a four-color standard filter set was employed with the DAPI channel used for CFW signal recording (excitation: 381–399 nm), GFP channel for GFP, and TRITC channel for mCherry (excitation: 529–566 nm).

For the quantification of the mCherry signal in the cell wall, a reference stain RSY5886 expressing Citrin was imaged together with the experimental strains having different promoter or mutations within their genomes and are identifiable by the presence of CFP in their cytosol. Images of life cells were captured with a Visitron VisiScope CSU-W1 (Visitron Systems, Puchheim, Germany). It consisted of a Nikon Ti-E inverted microscope, equipped with a CSU-W1 spinning disk head with a 50-µm pinhole disk (Yokogawa, Tokyo, Japan), with a scientific grade 4.2 sCMOS camera, and a PLAN APO 100× NA 1.3 oil immersion objective (Nikon). Twenty-eight optical sections with a step size of 300 nm were automatically analyzed to quantify mCherry signal in the cell wall. To do so different macros were written: (I) to sum up mCherry, Citrin and CFP signals, (II) to segment yeast cells, (III) to segment the cell wall, (IV) to compute pixel numbers, CFP, Citrin, mCherry minimal, maximal and integral intensity in the cell wall and within the cell. Only cells having an internal volume between 2400 and 55,000 pixels (empirically assigned value after analyzing three control images) were analyzed, and identity was assigned by analyzing maximal intensity within the cell (CFP >200, Citrin <200 for the reference stain and Citrin >200, CFP <200 for tester strains). Mean intensity of mCherry in the cell wall was calculated by dividing integral mCherry intensity in the cell wall by the number of pixels in cell wall. The workflow is presented in Fig. 6A. Images were processed and analyzed using FIJI software (Schindelin et al., 2012). The macros are available upon request.

### GFP labelling of Pry3-VHH expressing cells and *α*-factor treatment

To label cells expressing Pry3-VHH with GFP, we produced conditioned YPD medium containing soluble GFP as follows: cells secreting GFP were grown in selective medium for 8 h and then shifted to YPD for overnight growth. The following day, yeast cells were removed by centrifugation (5 min, 3000 rpm), and the medium was mixed with fresh YPD (a quarter of the volume). In parallel cells expressing Pry3-VHH, Pry3^E96A^-VHH or the empty vector were cultivated overnight in selective medium. The next morning six OD_600_ of cells were collected and diluted in 20 ml of the conditioned GFP containing YPD medium and cells were grown for an additional 4 h (Fig. 8B).

To induce shmoo formation, we followed a protocol adapted from (Breeden, 1997). Briefly, *MATa* cells were grown overnight in YPD to an OD_600_<0.8, washed with pre-warmed water and resuspended in pre-warmed YPD at a density of OD_600_=0.2-0.25. *α*-factor diluted in ethanol (stock solution 2 mg/ml) (KareBay Biochem, NJ, USA; kind gift of C. De Virgilio, University of Fribourg) was added at a final concentration of 20 µl/ml. Cells were collected and imaged 2 h after treatment.

### Calcofluor-white staining

Calcofluor-white staining was performed essentially as described previously (Pringle, 1991). 1 ml of yeast cells were collected, washed with water and resuspended in 1 ml freshly diluted (1:5) calcofluor-white solution (F-3543, Sigma-Aldrich, stock solution 1 mg/ml, stored at 4°C). Cells were then incubated 30 min at 30°C, washed three times with water, and finally resuspended in 50 µl of YPD before imaging.

## Supplementary Material

Supplementary information
