# Supplementary material for: The yeast cell wall protein Pry3 inhibits mating through highly conserved residues within the CAP domain
Source: Biol Open. 2020 Jul 3;9(6):bio053470. doi: 10.1242/bio.053470 (PMC7340583; doi:10.1242/bio.053470)
Supplement: Supplementary information [file biolopen-9-053470-s1.pdf]

Supplementary Materials

Supplementary Tables

Table S1. Yeast strains used in this study

Strain	Genotype	Source	Figure
BY4741	*MATa*; *his3Δ1, leu2Δ0, met15Δ0, ura3Δ0*	Euroscarf	1, 2B, 3C, 4B, 5, 7E, 7F, 8
BY4742	*MATα; his3Δ1, leu2Δ0, lys2Δ0, ura3Δ0*	Euroscarf	1, 2B, 3C, 4B, 5, 7F, 8
RSY6232	*[BY4741] HSP150-MYC::HIS3MX6*	This study	1C, 1D
pry3Δ a	*[BY4741] pry3Δ::kanMX4*	Euroscarf	1E
pry3Δ α	*[BY4742] pry3Δ::kanMX4*	Euroscarf	1E
RSY5858	*[BY4741] pry3ΔS169-T242::URA3*	This study	
RSY5872	*[BY4741] PRY3-mCHERRY*	This study	6B, 7
RSY5886	*[RSY5872] URA3::ADH1*^{prom}*::PRY3-mCHERRY*	This study	6, 7
RSY6513	*[RSY5886] URA3::ADH1*^{prom}*::PRY3*^{H113A}*-mCHERRY*	This study	6B
RSY6516	*[RSY5886] URA3::ADH1*^{prom}*::PRY3*^{C142S}*-mCHERRY*	This study	6B
RSY6538	*[RSY5886] URA3::ADH1*^{prom}*::PRY3*^{E96A}*-mCHERRY*	This study	6B
RSY5885	*[BY4742] PRY3-mCHERRY*	This study	
RSY5901	*[RSY5885] URA3::ADH1*^{prom}*::PRY3-mCHERRY*	This study	
Fus1-GFP	*[BY4741] ycl027w-GFP::HIS3MX6*	Huh et al., 2003	7C
RSY6140	*MATa*; *his3Δ1, leu2Δ0, lys2Δ0, met15Δ0, ura3Δ0, ycl027w-GFP::HIS3MX6, URA3::ADH1*^{prom}*-PRY3-mCHERRY*	This study	7C
RSY6144	*MATa; his3Δ1, leu2Δ0, met15Δ0, ura3Δ0, ycl027w-GFP::HIS3MX6, PRY3-mCHERRY*	This study	7C
Mid2-GFP	*[BY4741] ylr332w -GFP::HIS3MX6*	Huh et al., 2003	7C
RSY6143	*MATa; his3Δ1, leu2Δ0, lys2Δ0, met15Δ0, ura3Δ0, ylr332w-GFP::HIS3MX6, URA3::ADH1*^{prom}*-PRY3-mCHERRY*	This study	7C
RSY6147	*MATa; his3Δ1, leu2Δ0, met15Δ0, ura3Δ0, ylr332w-GFP::HIS3MX6 PRY3-mCHERRY*	This study	7C

Biology Open • Supplementary information

Table S2. Plasmids used in this study

Plasmids	Genotype	Source	Figure
pRS415	*CEN/ARS, ADH1^{prom}, LEU2*	Mumberg et al., 1995	1, 2B, 3C, 4B, 5B, 7F, 8
pRS426	2μ, *ADH1^{prom}, URA3*	Mumberg et al., 1995	1, 2B, 3C, 4B, 5B, 7F, 8B
p1314	[pRS426] *ADH1^{prom}-PRY1*	This study	1B
p1311	[pRS426] *ADH1^{prom}-PRY2*	This study	1B
p1123	[pRS426] *ADH1^{prom}-PRY3*	This study	1, 2B, 3C, 4B, 5B, 8B
p1907	[pRS426] *ADH1^{prom}-PRY1-HA*	This study	1C, 1D
p1908	[pRS426] *ADH1^{prom}-PRY2-HA*	This study	1C, 1D
p1314	[pRS426] *ADH1^{prom}-PRY3-HA*	This study	1C, 1D
	pFA6a-13Myc-His3MX6	(Longtine et al., 1998)	
p1206	[pRS426] *ADH1^{prom}-PRY3, LEU2*	This study	1E
p1124	[pRS426] *ADH1^{prom}-PRY3-ΔGPI*	This study	1F
p1126	[pRS426] *ADH1^{prom}-PRY3-CAP*	This study	1F
p1137	[pRS426] *ADH1^{prom}-PRY3-S/T*	This study	1F
p1136	[pRS426] *ADH1^{prom}-PRY3-ΔS/T*	This study	1F
p1138	[pRS426] *ADH1^{prom}-PRY3-ssXmaI*	This study	
p1334	[pRS426] *ADH1^{prom}-PRY3-ΔCAP*	This study	1F
p1254	[pRS426] *ADH1^{prom}-PRY3$^{CAP\text{-}Pry1}$*	This study	2B
p1361	[pRS426] *ADH1^{prom}-PRY3$^{CAP\text{-}Pry2}$*	This study	2B
pET22b	*PelB, C-terminal His-tag, AmpR*	Novagen	
PRY1^{CAP}-HA	[pET22-b] *PRY1-CAP*	Choudhary et al., 2014	3B, 3D
p1287	[pET22-b] *PRY3-CAP*	This study	3B, 3D
p1336	[pET22-b] *PRY3^{C142S}-CAP*	This study	3B
p1351	[pET22-b] *PRY3$^{P105C, A155C}$-CAP*	This study	3B
p1366	[pET22-b] *PRY3^{V117M}-CAP*	This study	3D
p1281	[pRS426] *ADH1^{prom}-PRY3^{C142S}*	This study	3C
p1306	[pRS426] *ADH1^{prom}-PRY3$^{P105C, A155C}$*	This study	3C
p1319	[pRS426] *ADH1^{prom}-PRY3^{V117M}*	This study	3C
p1516	[pRS426] *ADH1^{prom}-PRY3^{H71A}*	This study	4B
p1518	[pRS426] *ADH1^{prom}-PRY3^{S72A}*	This study	4B
p1517	[pRS426] *ADH1^{prom}-PRY3^{E78A}*	This study	4B
p1510	[pRS426] *ADH1^{prom}-PRY3^{E96A}*	This study	4B

Biology Open • Supplementary information

p1511	[pRS426] *ADH1*^{prom}-*PRY3*^{S103A}	This study	4B
p1512	[pRS426] *ADH1*^{prom}-*PRY3*^{H113A}	This study	4B
p1202	[pRS426] *ADH1*^{prom}-*PRY3-AAXmaI*	This study	
pGREG506	*CEN/ARS, GAL1*^{prom} *URA3*	Jansen et al., 2005	
pBS34	mCherry	Hailey et al., 2002	
p1142	[pGREG506] *PRY3*^{prom}-*PRY3-PRY3*$^{3'\text{-}end}$	This study	
p1245	[pRS426] *ADH1*^{prom}-*PRY3-mCHERRY*	This study	5B, C
p1283	[pGREG506] *PRY3*^{prom}-*PRY3-mCHERRY-PRY3*$^{3'\text{-}end}$	This study	5B, C
p1302	[pRS426] *ADH1*^{prom}-*PRY3*^{C142S}-*mCHERRY*	This study	5B, C
p1810	[pRS426] *ADH1*^{prom}-*PRY3*^{E96A}-*mCHERRY*	This study	5B, C
p1811	[pRS426] *ADH1*^{prom}-*PRY3*^{H113A}-*mCHERRY*	This study	5B, C
p1309	[pGREG506] *PRY3*^{prom}-*PRY3*^{C142S}-*mCHERRY-PRY3*$^{3'\text{-}end}$	This study	5B, C
p1812	[pGREG506] *PRY3*^{prom}-*PRY3*^{E96A}-*mCHERRY-PRY3*$^{3'\text{-}end}$	This study	5B, C
p1813	[pGREG506] *PRY3*^{prom}-*PRY3*^{H113A}-*mCHERRY-PRY3*$^{3'\text{-}end}$	This study	5B, C
pRCC-K	2μ, *ROX3p-cas9-CYC1*^{ter}, *SNR52p-gRNA, kanMX*	Generoso et al., 2016	
p1918	[pRCC-K] target sequence (3'-CAAATCCCGGATTTTCTGAATCCAC-5')	This study	
p1919	[pRCC-K] target sequence (3'-GAGCGGTGGACGCCTGGTACG-5')	This study	
p1920	[pRCC-K] target sequence (3'-GTGTGCTCCTACAACCCTCC-5')	This study	
pKT174	[pFA6a] *link-yECFP, CaURA3*	Sheff and Thorn, 2004	
p1174	[pRS415] *ADH1p-yECFP*	This study	6
pKT211	[pFA6a] *link-yEmCitrine, SpHIS5*	Sheff et al., 2004	
p1921	[pRS415] *ADH1p- yEmCitrine*	This study	6
pRS425	2μ, *ADH1*^{prom}, *LEU2*	Mumberg et al., 1995	
p2040	[pRS425] *ADH1*^{prom}-*PRY3*^{ss}-*PRY3*^{CAP}-*GFP-CCW12*	This study	7E
p2041	[pRS425] *ADH1*^{prom}-*PRY3*^{ss}-*PRY3*$^{E96A,\ CAP}$-*GFP-CCW12*	This study	7E

pRH2776	[pRS413] *VAC8-vhhGFP4*	Powis et al., 2015	
p1260	[pRS426] *ADH1^{prom}-PRY3-VHH*	This study	
p1906	[pRS426] *ADH1^{prom}-PRY3^{E96A}-VHH*	This study	
pGREG503	*CEN/ARS, GAL1^{prom}, HIS3*	Jansen et al., 2005	
KAR2ss-CFP-HDEL	[pRS315] *PGK1^{prom}-KAR2^{ss}-CFP-HDEL*	Szymanski et al., 2007	
pKT128	[pFA6a] *link-yEGFP, SpHIS5*	Sheff et al., 2004	
p1719	[pRS315] *PGK1^{prom}-KAR2^{ss}-GFP*	This study	8D, E, F
p1933	[pRS315] *PGK1^{prom}-PRY3^{ss}-PRY3^{CAP}-GFP*	This study	8F
p1934	[pRS315] *PGK1^{prom}-PRY3^{ss}-PRY3$^{E96A, CAP}$-GFP*	This study	8F

References

Choudhary, V., Darwiche, R., Gfeller, D., Zoete, V., Michielin, O. and Schneiter, R. (2014). The caveolin-binding motif of the pathogen-related yeast protein Pry1, a member of the CAP protein superfamily, is required for in vivo export of cholesteryl acetate. *J Lipid Res.* **55,** 883-894.

Generoso, W. C., Gottardi, M., Oreb, M. and Boles, E. (2016). Simplified CRISPR-Cas genome editing for Saccharomyces cerevisiae. *J Microbiol Methods.* **127,** 203-205.

Hailey, D. W., Davis, T. N. and Muller, E. G. (2002). Fluorescence resonance energy transfer using color variants of green fluorescent protein. *Methods Enzymol.* **351,** 34-49.

Huh, W. K., Falvo, J. V., Gerke, L. C., Carroll, A. S., Howson, R. W., Weissman, J. S. and O'Shea, E. K. (2003). Global analysis of protein localization in budding yeast. *Nature.* **425,** 686-691.

Jansen, G., Wu, C., Schade, B., Thomas, D. Y. and Whiteway, M. (2005). Drag&Drop cloning in yeast. *Gene.* **344,** 43-51.

Longtine, M. S., McKenzie, A., Demarini, D. J., Shah, N. G., Wach, A., Brachat, A., Philippsen, P. and Pringle, J. R. (1998). Additional modules for versatile and economical PCR-based gene deletion and modification in Saccharomyces cerevisiae. *Yeast.* **14,** 953-961.

Mumberg, D., Muller, R. and Funk, M. (1995). Yeast vectors for the controlled expression of heterologous proteins in different genetic backgrounds. *Gene.* **156,** 119-122.

Powis, K., Zhang, T., Panchaud, N., Wang, R., De Virgilio, C. and Ding, J. (2015). Crystal structure of the Ego1-Ego2-Ego3 complex and its role in promoting Rag GTPase-dependent TORC1 signaling. *Cell Res.* **25,** 1043-1059.

Biology Open • Supplementary information

Sheff, M. A. and Thorn, K. S. (2004). Optimized cassettes for fluorescent protein tagging in Saccharomyces cerevisiae. *Yeast.* **21,** 661-670.

Szymanski, K. M., Binns, D., Bartz, R., Grishin, N. V., Li, W. P., Agarwal, A. K., Garg, A., Anderson, R. G. and Goodman, J. M. (2007). The lipodystrophy protein seipin is found at endoplasmic reticulum lipid droplet junctions and is important for droplet morphology. *Proc Natl Acad Sci U S A.* **104,** 20890-20895.

Biology Open • Supplementary information
